# Proportion of serum thyroid hormone concentrations within the reference ranges in athyreotic patients on levothyroxine monotherapy: a retrospective study

**DOI:** 10.1186/s13044-022-00127-3

**Published:** 2022-05-10

**Authors:** Mitsuru Ito, Sawako Takahashi, Mikiko Okazaki-Hada, Mizuho Minakata, Kazuyoshi Kohsaka, Tomohiko Nakamura, Toshihiko Kasahara, Takumi Kudo, Eijun Nishihara, Shuji Fukata, Mitsushige Nishikawa, Takashi Akamiuzu, Akira Miyauchi

**Affiliations:** Kuma Hospital, Center for Excellence in Thyroid Care, 8-2-35 Shimoyamate-Dori, Chuo-Ku, Kobe, Hyogo 650-0011 Japan

**Keywords:** Thyroxine, Triiodothyronine, Thyroid-stimulating hormone suppressive therapy, Athyreotic patients

## Abstract

**Background:**

In patients receiving thyroid-stimulating hormone (TSH) suppressive therapy with levothyroxine (LT_4_) after total thyroidectomy for thyroid cancer, thyroid function tests should be performed to adjust the LT_4_ dose. Specifically, serum TSH concentrations are commonly measured because TSH suppression is necessary according to thyroid cancer risk. The aim of the present study was to elucidate whether free thyroxine (FT_4_) or free triiodothyronine (FT_3_) indicates better for adjusting the dose in athyreotic patients on LT_4_ monotherapy after total thyroidectomy.

**Methods:**

We retrospectively studied the compatibility of free thyroid hormone (FT_4_ and FT_3_) concentrations with reference ranges in athyreotic patients on LT_4_ monotherapy after total thyroidectomy.

**Results:**

We identified 2210 consecutive patients from their medical records. Of these patients, 250 had both FT_4_ and FT_3_ concentrations in addition to TSH. Two hundred seven had serum TSH concentrations below the reference range (0.5–5.0 μIU/mL), while 43 had them within the reference range. In the 207 patients with TSH concentrations below the reference range, 61 patients (29.5%) had FT_4_ concentrations within the reference range (0.9–1.7 ng/dL) and 146 patients (70.5%) had FT_4_ concentrations above the reference range. In contrast, 10 patients (4.8%) had FT_3_ concentrations below the reference range (2.3–4.0 pg/mL) and 8 (3.9%) had FT_3_ concentrations above the reference range; 189 patients (91.3%) had concentrations within the reference range. Of the 43 patients with TSH concentrations within the reference range, 25 (58.1%) had FT_4_ concentrations within the reference range and 18 (41.9%) had FT_4_ concentrations above the reference range. While, 11 patients (25.6%) had FT_3_ concentrations below the reference range and one (2.3%) had FT_3_ concentrations above the reference range; hence, 31 patients (72.1%) had FT_3_ concentrations within the reference range.

**Conclusion:**

This study showed that measuring FT_3_ concentrations rather than FT_4_ concentrations as the subsequent parameter of thyroid function might be more useful for disease management in terms of the proportion of serum thyroid hormone concentrations within the reference ranges. Furthermore, FT_3_ measurement could be useful in providing more detailed treatments, including avoiding more aggressive TSH suppressive therapy and identifying the presence of low T_3_ syndrome in the background.

## Background

There are two thyroid hormones, thyroxine (T_4_) and triiodothyronine (T_3_). T_3_ is the biologically active thyroid hormone. In normal subjects, 100% of T_4_ and approximately 20% of T_3_ are secreted by the thyroid gland, and approximately 80% of T_3_ is derived from the conversion of T_4_ to T_3_ in extra-thyroidal peripheral tissues [1]. Thus, a relative T_3_ deficiency may be present in athyreotic patients during levothyroxine (LT_4_) monotherapy. We and other investigators [2–4] compared postoperative T_3_ concentrations in patients on LT_4_ therapy with their preoperative concentrations or concentrations in euthyroid controls and observed that among athyreotic patients who underwent total thyroidectomy and received LT_4_, patients with normal serum thyroid-stimulating hormone (TSH) concentrations had mildly low serum-free triiodothyronine (FT_3_) concentrations, patients with mildly suppressed serum TSH concentrations had normal serum FT_3_ concentrations, and patients with strongly suppressed serum TSH concentrations had increased serum FT_3_ concentrations. Serum-free thyroxine (FT_4_) concentrations were significantly increased in all groups; however, the magnitude of the increase varied according to the TSH concentration.

Thyroid function tests are used in several clinical settings to evaluate thyroid dysfunction, assess the adequacy of LT_4_ therapy, and monitor hyperthyroidism treatment. Patients with primary hypothyroidism who are receiving LT_4_ can be monitored by assessing serum TSH concentrations because serum FT_4_ measurements lack sensitivity to assess the appropriateness of the LT_4_ dose. In general, patients with hypothyroidism who are receiving LT_4_, including TSH suppressive therapy, can be monitored by assessing serum TSH and FT_4_ concentrations [5]. While several investigators reported that athyreotic patients on LT_4_ had relatively high FT_4_ concentrations and comparable FT_3_ concentrations compared to preoperative values, suggesting that FT_3_ measurements may be more useful than FT_4_ measurements in adjusting thyroid hormone replacement therapy in such patients when referring to reference values in healthy subjects [2–4].

This aim of study is to see whether athyretotic patients on LT_4_ monotherapy after total thyroidectomy are more likely to have either FT_4_ or FT_3_ measurements within reference intervals when the therapeutic goal is to maintain TSH within or below the reference range and elucidate whether FT_4_ or FT_3_ is a better indicator for adjusting the dose in such patients. To facilitate this study, only patients with papillary thyroid carcinoma without relevance to the thyroidal conversion of T_4_ to T_3_ [6] were selected.

## Methods

From their medical records, we identified 2210 consecutive patients who underwent a thyroidectomy for papillary thyroid carcinoma between January 2019 and March 2021 at Kuma Hospital and were followed at least for 6 months postoperatively. Among 2210 patients, TSH and FT_4_ levels were measured in 511 patients using thyroid function tests and TSH and FT_3_ levels were measured in 1449 patients using thyroid function tests. Two hundred fifty patients had their TSH, FT_4_, and FT_3_ levels measured. In the present study, we evaluated the compatibility of free thyroid hormone (FT_4_ and FT_3_) concentrations with reference ranges in these 250 patients who had both FT_4_ and FT_3_ measurements. The following patients were excluded: (1) those who underwent near-total or subtotal thyroidectomy; (2) those with thyroid malignancies besides papillary carcinoma; (3) those with thyroid dysfunction, including Graves’ disease, thyroid dyshormonogenesis, or autonomously-functioning thyroid nodules; (4) those whose medications, including amiodarone, lithium, β-blocker, or iodine-containing drugs, directly affected thyroid function; or (5) those who were pregnant or lactating. Patients who had postsurgical hypoparathyroidism and those who failed to achieve suppression of TSH concentrations were also excluded. The included patients who underwent total thyroidectomy were initially administered 2.0 μg/kg LT_4_ daily after surgery. Thyroid function tests were performed 1 month after surgery and every 2–3 months thereafter. The LT_4_ dosage was adjusted to achieve the target TSH levels determined according to the risk of recurrence based on the three-level stratification in American Thyroid Association (ATA) guidelines [7]. The target serum TSH levels were strongly suppressed TSH levels (≤ 0.05 lIU/mL) for the high-risk patients, mildly suppressed TSH levels (0.05 < TSH ≤ 0.5 lIU/mL) for the intermediate-risk patients, and normal TSH levels (0.5 < TSH ≤ 5 lIU/mL) for the low-risk patients. The present study was approved by the Ethical Committee at Kuma Hospital (No 20200709–1), and all patients provided informed consent.

### Thyroid function tests

Postoperative thyroid profiles of each patient were obtained after stabilizing the thyroid profiles for at least 6 months after thyroidectomy. Blood samples were obtained in the morning after the patient fasted overnight and after ingesting LT_4_. Serum TSH, FT_4_, and FT_3_ concentrations were measured using an electrochemiluminescence immunoassay (Elecsys; Roche Diagnostics GmbH, Mannheim, Germany). Reference ranges were calculated using samples from healthy Japanese adult volunteers [8]. The reference ranges for TSH were calculated from Mean ± 2SD of lognormal distribution using serum from 824 subjects (0.5–5.0 μIU/mL). The reference ranges for FT4 were calculated from the 95% range by non-parametric method using serum from 738 subjects (0.9–1.7 ng/dL). The reference ranges for FT3 were calculated from the 95% range by non-parametric method using serum from 838 subjects (2.3–4.0 pg/mL). The intra-assay coefficients of variation were ≤ 10% for the TSH assay, ≤8% for the FT_4_ assay, and ≤ 10% for the FT_3_ assay.

### Statistical analysis

Grouped data were expressed as the mean ± standard deviation or the median (25th to 75th percentiles). Postoperative two-group comparisons were performed using the χ^2^ test (gender), unpaired t-test in case of normal distribution, or Mann-Whitney *U* test in case of nonparametric distribution. Significance was defined with two-sided *p-*values < 0.05. Statistical analyses were performed using the StatFlex version 6.0 (Artech Co., Ltd., Osaka, Japan).

## Results

### Characteristics of the two groups in which FT_4_ or FT_3_ concentrations were measured using thyroid function tests

Among 250 patients, 207 had serum TSH concentrations below the reference range (0.5–5.0 μIU/mL) (Group I) and 43 had them within the normal range (Group II). In the present study, we examined the compatibility of each thyroid hormone measurement (FT_4_ or FT_3_) with the reference range in the two patient groups. The characteristics of patients in the two groups are shown in Table 1.

**Table 1:** Clinical characteristics in the two patient groups with suppressed TSH levels (I) and normal TSH levels (II)

Patient Subgroups	Group I	Group II	p^a^
No of patients (male)	207 (35)	43 (8)	ns
Age (years)	53 ± 16	61 ± 17	< 0.01
Follow-up time (day)	2914 ± 1649	3406 ± 1563	ns
LT_4_ dose (μg/day)	125 (100–150)	125 (100–137.5)	ns*
TSH (μIU/mL)	0.027 (0.009–0.095)	1.190 (0.909–2.395)	< 0.001*
FT_4_ (ng/dL)	1.91 (1.67–2.16)	1.63 (1.46–1.80)	< 0.001
FT_3_ (pg/mL)	3.10 (2.74–3.45)	2.64 (2.31–2.82)	< 0.001

^a^Statistical significance was analyzed by the χ^2^ test (sex), unpaired t-test, or *Mann–Whitney U test. Values are expressed as mean ± SD or median (25th–75th percentiles)

*Abbreviations*: *TSH* Thyroid stimulating hormone, *LT*_*4*_ Levothyroxine, *FT*_*4*_ Free thyroxine, *FT*_*3*_ Free triiodothyronine

### Compatibility of FT_4_ or FT_3_ concentrations measured through thyroid function tests with reference ranges in patients with suppressive serum TSH concentrations

**Fig. 1:**
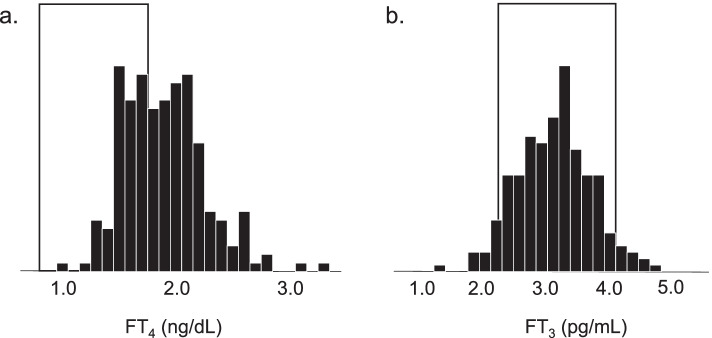
Compatibility of FT_4_ (**A**) or FT_3_ (**B**) concentrations measured through thyroid function tests with reference ranges in patients with suppressive serum TSH concentrations*.* Among 250 patients, 207 had serum TSH concentrations below the reference range (0.5–5.0 μIU/mL). The reference range for each thyroid hormone is indicated by the square area. Among 207 patients, 61 patients (29.5%) had FT_4_ concentrations within the reference range (0.9–1.7 ng/dL) and 146 (70.5%) had FT_4_ concentrations above the reference range. However, none of them had serum FT_4_ concentrations below the reference range. On the other hand, 10 patients (4.8%) had FT_3_ concentrations below the reference range (2.3–4.0 pg/mL) and 8 patients (3.9%) had FT_3_ concentrations above the reference range; hence, 189 patients (91.3%) had concentrations within the reference range. FT_4_, free thyroxine; FT_3,_ free triiodothyronine

Figure 1 shows the compatibility of FT_4_ (A) or FT_3_ (B) concentrations with reference ranges in patients with suppressive serum TSH concentrations. Of the 207 patients, 61 (29.5%) had FT_4_ concentrations within the reference range (0.9–1.7 ng/dL) and 146 (70.5%) had FT_4_ concentrations above the reference range. However, none of them had serum FT_4_ concentrations below the reference range. In contrast, 10 patients (4.8%) had FT_3_ concentrations below the reference range and 8 (3.9%) had FT_3_ concentrations above the reference rang; hence, 189 patients (91.3%) had FT_3_ concentrations within the reference range (2.3–4.0 pg/mL).

### Compatibility of FT_4_ or FT_3_ concentrations measured through thyroid function tests with reference ranges in patients with normal serum TSH concentrations

**Fig. 2:**
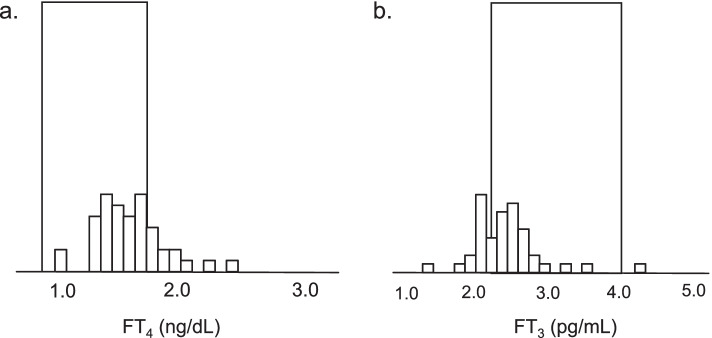
Compatibility of FT_4_ (**A**) or FT_3_ (**B**) concentrations measured through thyroid function tests with reference ranges in patients with normal serum TSH concentrations*.* Among 250 patients, 43 had them within the normal range (0.5–5.0 μIU/mL). The reference range for each thyroid hormone is indicated by the square area. Among 43 patients, 25 patients (58.1%) had FT_4_ concentrations within the reference range (0.9–1.7 ng/dL) and 18 (41.9%) had FT_4_ concentrations above the reference range. However, none of them had serum FT_4_ concentrations below the reference range. On the other hand, 11 patients (25.6%) had FT_3_ concentrations below the reference range (2.3–4.0 pg/mL) and one patient (2.3%) had FT_3_ concentrations above the reference range; hence, 31 patients (72.1%) had concentrations within the reference range. FT_4_, free thyroxine; FT_3,_ free triiodothyronine

Figure 2 shows the compatibility of FT_4_ (A) or FT_3_ (B) concentrations with reference ranges in patients with normal serum TSH concentrations. Of the 43 patients, 25 (58.1%) had FT_4_ concentrations within the reference range (0.9–1.7 ng/dL) and 18 (41.9%) had FT_4_ concentrations above the reference range. However, none of them had FT_4_ concentrations below the reference range. In contrast, 11 patients (25.6%) had FT_3_ concentrations below the reference range and one (2.3%) had FT_3_ concentrations above the reference range; hence, 31 patients (72.1%) had FT_3_ concentrations within the reference range (2.3–4.0 pg/mL).

### Serum FT_3_ concentrations above or below the reference range in athyreotic patients receiving TSH suppressive therapy

We further investigated athyreotic patients receiving TSH suppressive therapy with abnormal FT_3_ concentrations. We found that 7 of 8 patients with FT_3_ concentrations above the reference range had completely suppressed TSH concentrations. Among 8 patients with FT_3_ concentrations above the reference range, the LT_4_ dose was reduced in 5 patients. In 6 of the 10 patients with FT_3_ concentrations below the reference range, the reduction of FT_3_ concentration was transient. In these patients, incidental poor compliance with LT_4_ medication and instability of measurement were suspected. Thus, 4 patients had persistently FT_3_ concentrations below the reference range; 3 of these 4 patients had underlying conditions: multiple lung and bone metastases, chronic renal failure, and low body mass index, which could be the cause of their reduction of serum T_3_ concentrations. Of these 4 patients, the LT_4_ dose was not increased in 3 patients with low T_3_ syndrome, while the dose was subsequently increased for the remaining one patient.

## Discussion

In this study, we examined the compatibility of FT_4_ or FT_3_ with reference ranges in athyreotic patients with suppressed or normal TSH concentrations treated with LT_4_ after total thyroidectomy for thyroid cancer.

In patients with TSH concentrations within the reference range, FT_4_ concentrations were above the reference range in just under half of them (41.9%), and FT_3_ concentrations were below the reference range in a quarter of them (25.6%). Regarding the compatibility of FT_4_ and FT_3_ concentrations with reference ranges in patients receiving LT_4_ after total thyroidectomy, Gullo et al. examined patients with normal serum TSH concentrations and reported that FT_4_ concentrations were above the reference range in 7.2% of patients and FT_3_ concentrations were below the reference range in 15.2% of patients. They concluded that FT_4_ and FT_3_ concentrations are not necessarily within reference ranges in patients with normal TSH concentrations [3]. Several studies, including ours, compared postoperative T_3_ concentrations in patients receiving LT_4_ therapy with their preoperative concentrations or with the concentrations in euthyroid controls [2, 4] and found that among athyreotic patients receiving LT_4_ after total thyroidectomy, those with normal serum TSH concentrations had mildly high serum FT_4_ concentrations, and mildly low serum FT_3_ concentrations; these results were coherent with those of the present study.

In patients with suppressed TSH concentrations, we found that serum FT_4_ concentrations were above the reference range in most patients (70.5%), whereas serum FT_3_ concentrations were within the reference range in most patients (91.3%). In our previous study [2, 9, 10], we reported that patients with mildly suppressed serum TSH concentrations had normal serum FT_3_ concentrations, and those with strongly suppressed serum TSH concentrations had increased serum FT_3_ concentrations. Serum FT_4_ concentrations were significantly increased in all groups; however, the magnitude of increase varied with TSH concentrations. Therefore, many patients receiving TSH suppressive therapy with LT_4_ after total thyroidectomy may have serum FT_4_ concentrations above the upper end of the reference range and serum FT_3_ concentrations within the reference range; these results were confirmed in the present study.

In the present study, serum TSH concentrations in patients with FT_3_ concentrations above the reference range were completely suppressed in the majority of cases. In fact, the LT_4_ dose was reduced in some patients with completely suppressed TSH concentrations and elevated FT_3_ concentrations. In such cases, the reduction of LT_4_ dosage may be considered reasonable, especially in patients with symptoms of thyrotoxicosis; those with suspected complications, such as osteoporosis or atrial fibrillation; or low-risk patients. Several studies have suggested that TSH suppressive therapy after total thyroidectomy for thyroid cancer might increase the risk of complications, such as osteoporosis [11] or atrial fibrillation [12]. However, the potential role of a different degree of TSH suppression for such complications remains to be established. Klein et al. reported a relationship between the degree of TSH suppression and cardiovascular disease mortality. In their study, both cardiovascular and all-cause mortality rates increased with complete TSH suppression but not with mild TSH suppression [13]. We reported that athyreotic patients receiving LT_4_ with mild TSH suppression and FT_4_ concentrations above the reference range but normal FT_3_ concentrations, metabolic indicators [9], and physical symptoms [10] were in a euthyroid state. In contrast, athyreotic patients with complete TSH suppression that resulted in both FT_4_ and FT_3_ concentrations above the reference range, metabolic indicators [9], and physical symptoms [10] were in a thyrotoxic state. These data suggest that among athyreotic patients receiving LT_4_, patients with mildly suppressed TSH and normal FT_3_ concentrations were closest to the euthyroid state, whereas those with completely suppressed TSH concentrations and FT_3_ concentrations above the reference range were in the thyrotoxic state. Therefore, in patients with TSH suppression receiving LT_4_ after total thyroidectomy, determination of serum FT_3_ and TSH concentrations may be useful to avoid thyrotoxicosis.

In the present study, the duration of abnormal concentrations was transient in patients with FT_3_ concentrations below the reference range, probably because of the variability of FT_3_ concentrations measured in trace amounts. However, some cases with persistently FT_3_ concentrations below the reference range were attributed to low T_3_ syndrome caused by underlying diseases. In such cases, an increase in the LT_4_ dose would have been inappropriate. In the case of FT_3_ concentrations below the reference range in TSH suppressive therapy after total thyroidectomy, it may be necessary to pay attention to the presence or absence of an underlying disease and monitor the persistence of FT_3_ concentrations below the reference range by repeated measurements to confirm the presence of a low T_3_ syndrome [14].

This study had some possible limitations. First, in this retrospective study, only patients who underwent both FT_4_ and FT_3_ measurements were included, while patients who underwent either FT_4_ or FT_3_ measurement because the simultaneous measurement of FT_4_ and FT_3_ would not be approved for insurance reimbursement were excluded. Only 250 out of the 2100 eligible subjects were studied in this study. Thus, this selection may have influenced the results. Second, we examined only the compatibility of thyroid hormones with the reference range; thus, further well-designed studies, including clinical features of thyroid hormone excess or deficiency, or the recurrence of thyroid cancer may be necessary.

## Conclusions

In the present study, we evaluated whether athyretotic patients on LT_4_ after total thyroidectomy are more likely to have either FT_4_ or FT_3_ measurements within reference intervals when the therapeutic goal is to maintain TSH within or below the reference range. As a result, the majority of patients with suppressed TSH concentrations had serum FT_4_ concentrations above the upper end of the reference range and serum FT_3_ concentrations within the reference range. While, fewer patients with TSH within the reference range had FT_3_ concentrations within the reference range. This study showed that measuring FT_3_ concentrations rather than FT_4_ concentrations as the subsequent parameter of thyroid function may be more useful for disease management in athyreotic patients on LT_4_ in terms of the proportion of serum thyroid hormone concentrations within the reference ranges. Furthermore, FT_3_ measurement could be useful in providing more detailed treatments, including avoiding more aggressive TSH suppressive therapy and identifying the presence of low T_3_ syndrome in the background.

## Data Availability

The datasets generated during and/or analysed during the current study are available from the corresponding author, Ito M, on reasonable request.

## Abbreviations

TSH: Thyroid-Stimulating Hormone
LT_4_: Levothyroxine
FT_4_: Free thyroxine
FT_3_: Free triiodothyronine
T_4_: Thyroxine
T_3_: Triiodothyronine
